# Pyrimidine and Purine Concentrations in Cord, Preterm and Term Infant Plasma Compared to Maternal Values—An Observational Study

**DOI:** 10.3390/nu18203351

**Published:** 2026-10-13

**Authors:** Michaela Minarski, Fee Rottach-Barnekow, Anna Shunova, Katrin Böckmann, Wolfgang Bernhard, Cornelia Wiechers, Christian F. Poets, Axel R. Franz

**Affiliations:** 1 Department of Neonatology, University Children’s Hospital, 72076 Tuebingen, Baden-Wuerttemberg, Germanyanna.shunova@med.uni-tuebingen.de (A.S.); wolfgang.bernhard@med.uni-tuebingen.de (W.B.); cornelia.wiechers@med.uni-tuebingen.de (C.W.); christian-f.poets@med.uni-tuebingen.de (C.F.P.); axel.franz@med.uni-tuebingen.de (A.R.F.); 2 Center for Pediatric Studies, University Children’s Hospital, 72076 Tuebingen, Baden-Wuerttemberg, Germany

**Keywords:** infant, preterm, very low birth weight, enteral feeding, nutrition, breast milk, purines, pyrimidines, micronutrients, conditionally essential nutrients

## Abstract

Background: Purines and pyrimidines are essential for the synthesis of nucleotides for DNA/RNA synthesis and for intermediate lipids, carbohydrates, and one-carbon metabolism. They are defined as nonessential as they can be synthesized de novo in vivo. However, the rate of de novo synthesis may be below requirements during rapid growth. Accordingly, nucleotide supplementation has been associated with potential benefits for growth in newborn infants. Therefore, nucleotides have been considered conditionally essential, and adequate availability may be relevant during periods of growth and development, particularly in preterm infants. Aims: To investigate plasma concentrations of purines and pyrimidines in cord blood in relation to (1) gestational age, (2) the blood of the corresponding mother, and (3) postnatal preterm infants’ blood. Methods: 155 infant–mother pairs (23 6/7–41 5/7 weeks postmenstrual age) from 10/2020–11/2022 were included. Purines and pyrimidines were analyzed with tandem mass spectrometry. Data are expressed as the median and interquartile range. Results: Cytidine, uridine, inosine, xanthine, hypoxanthine, adenosine, adenosine monophosphate, and guanosine monophosphate concentrations were above the lower limits of detection. Fetal plasma concentrations were significantly higher than those in corresponding mothers. The more immature the fetuses, the higher were the concentrations in umbilical cord plasma. Cytidine, inosine, xanthine, and hypoxanthine plasma concentrations significantly decreased within 14 days after birth. Conclusions: Fetal plasma concentrations of several purines and pyrimidines were higher than corresponding maternal concentrations, and concentrations of several metabolites decreased markedly after birth, demonstrating substantial changes in purine and pyrimidine concentrations across the fetal-to-postnatal transition. The underlying mechanisms (including potential contributions of placental transport, endogenous metabolism, tissue uptake, elimination, and nutritional intake) require further investigation.

## 1. Introduction

Pyrimidines and purines are heterocyclic compounds, comprising the nucleobases uracil, cytosine, and thymine, as well as adenine and guanine. Attached to the five-carbon sugars ribose or deoxyribose, they are called (deoxy)nucleosides, and further phosphorylation results in (deoxy)nucleotides. Pyrimidines and purines are essential for all living organisms in the form of deoxyribonucleic acid (DNA) as the genetic code and as ribonucleic acid (RNA) for protein synthesis. Hence, purines and pyrimidines are essential to parenchymal growth. However, they additionally play central roles in many metabolic pathways: adenosine, as a purine derivative, is part of S-adenosyl-methionine, representing the most important methyl group donor of the organism [1]. Beyond this, cytidine triphosphate (CTP) is essential to the synthesis of practically all membrane phospholipids, either via cytidylation of the phospholipid headgroup or that of the diacylglycerol unit. Moreover, guanosine and uridine triphosphate (GTP, UTP) are essential for carbohydrate metabolism (UDP-glucose, GDP-mannose) [2]. Finally, adenosine in adenosine triphosphate (ATP) is essential as a ubiquitous cellular energy currency [3].

Purines and pyrimidines can be synthesized de novo; are recycled via salvage pathways from the breakdown of DNA, RNA, and other nucleotide-containing compounds; and can partly be interconverted. Due to their pathway of synthesis, they are not regarded as essential [4]. However, when metabolic requirements are beyond the capacity of synthesis, as, e.g., during rapid fetal and preterm infant growth, any compound becomes conditionally essential [5]. This has already been demonstrated for other endogenously synthesizable substances, namely arachidonic acid, docosahexaenoic acid, and choline [6,7], and may similarly apply to purines and pyrimidines. This claim is substantiated by the findings that nucleotide supplementation leads to improved intestinal growth [8] and repair [9] and increased iron uptake [10] in animal models, better growth in small-for-gestational-age (SGA) infants [11], and better growth [12], as well as higher concentrations of long-chain polyunsaturated fatty acids in plasma and erythrocyte membrane phospholipids of full-term infants [13,14]. In addition, it has multiple positive effects on the immune system [15] of term infants. Hence, it might generally improve parenchymal growth and development in SGA and preterm infants [16], in whom parenchymal (i.e., lean body mass) growth is substantially impaired [17], but current evidence is still pending.

Human breast milk is considered the ideal nutrition for term and preterm infants [18]. Twenty-five percent of total nitrogen in breast milk is non-protein nitrogen (NPN) including that from nucleobases, -sides, and -tides [19,20]. Dominated by nucleotides, their concentrations and composition in breast milk vary inter-individually, regionally, and during lactation, but generally more nucleotides are found in human than in bovine milk [20]. In human milk, nine nucleotides were found (UMP (179 (±19.8) µg/100mL), UDP (174 (±12.8) µg/100mL), GMP (138 (± 8.5 µg/100mL), GDP (96 (±8.9) µg/100mL), CMP (461 (±17.9) µg/100mL), CDP (474 (±41.4) µg/100mL), AMP (175 (±12.8) µg/100mL), ADP (69 (±17.9) µg/100mL), IMP (228 (±14.5) µg/100mL), while only two were found in cow milk in low concentrations (AMP: 111 µg/100mL; ADP: 38 µg/100mL) [20]. To address this difference, nucleotides are commonly added to formulas for term and preterm infants [21,22]. However, for preterm infants, even nutrient supply via breast milk is insufficient to meet their 3–4-fold higher growth rate compared to term infants [23], which may also apply to nucleotides.

Consequently, if a diet adhering to current guidelines leads to an undersupply of premature infants with pyrimidines and purines, supplementation may improve their health positively in many different ways [16].

This observational study aims to investigate the concentrations of purines and pyrimidines in preterm and term infant plasma, compared to that of age-matched fetuses (cord blood), their mothers, and pregnant women in order to estimate the adequacy of current nutritional status of preterm infants.

## 2. Materials and Methods

### 2.1. Study Population

This observational study was performed from October 2020 to November 2022 at Tübingen University Hospital, Germany. It was registered with clincaltrials.gov (NCT03532633), and the protocol was approved by the Institutional Ethics Committee. Written informed parental consent was obtained, and the study was conducted in accordance with the Declaration of Helsinki. We aimed to include 150 infants and their mothers, divided into 5 gestational age groups at 30 patients each (23 0/7–27 6/7 weeks; 28 0/7–31 6/7 weeks; 32 0/7–34 6/7 weeks; 35 0/7–36 6/7 weeks; 37 0/7–41 6/7 weeks). Infants with malformations or syndromic and metabolic disorders were excluded from the study.

### 2.2. Study Design (Prospective Observational Study) and Timing of Blood Samples

At least 200 µL of venous EDTA blood samples were collected from the umbilical cord of potential study candidates, centrifuged, and plasma frozen immediately after birth. If the mother declined participation, the umbilical cord blood sample was discarded. If parental consent was obtained, 2.7 mL of EDTA-blood were collected from mothers within 48 h of birth. In addition, 200 µL of EDTA-blood was collected at 3 ± 1 and 14 ± 2 days (d), respectively, of postnatal age, as part of the routine blood draws required by our neonatal unit’s standards. If the study participant had already been discharged home by that time, the corresponding blood draws were not performed. As maternal blood sampling was predominantly performed postpartum (only two prepartum samples), eight additional women were subsequently included for prepartum blood sampling, resulting in a total of ten prepartum samples, to exploratively assess potential differences in purine and pyrimidine concentrations related to the timing of sampling relative to delivery.

Relevant clinical data were extracted from maternal and infant charts, including gestational age (GA) at birth, birth weight, maternal age, parity, mode of delivery, and nutritional data of infants during NICU stay. Our standard feeding protocol aims for rapid progression to enteral feeding for all preterm infants. For infants weighing <2500 g, not only parenteral glucose but also amino acids are provided during the introduction of oral feeds; for infants < 32 weeks or <1500 g, parenteral fat is administered additionally. To document any deviations, the exact nutrient intake (parenteral/enteral) was recorded on the day before the first blood draw, as well as during the five days prior to the second blood draw.

### 2.3. Chemical Analysis

Chemicals: Chloroform from Baker (Gliwice, Poland) was HPLC grade. Methanol and acetonitrile (Carl Roth GmbH, Karlsruhe, Germany) were analytical grade. Deionized water was obtained from a Milli-Q system (Merck Millipore, Darmstadt, Germany). Adenine, adenine-1,3-15N2, adenosine-15N5,5-monophosphat, adenosine-15N5,5 monophosphate sodium salt, adenosine-5′-monophosporicacid monohydrate, cytidine, cytidine 5′-monophosphoric disodium salt, cytosine-1,4-13C2, guanine, guanosine, guanosine 5′-monophosporic disodium hydrate, hypoxanthine, inosine, inosine 5′monophosporic disodium hydrate, thymidine, thymidine 5′monophosporic disodium hydrate, thymine, uracile, uridine, uridine 5′monophosporic disodium hydrate and xanthine were from Sigma Aldrich Chemie GmbH (Steinheim, Germany), while uridine-5,6-d2 was from CDN Isotopes (Pointe-Claire, QC, Canada). Purity of chemicals was checked by tandem mass spectrometry (see below). All other chemicals were of analytical grade and from various commercial sources.

#### 2.3.1. Plasma Samples

##### Sample Collection

EDTA blood samples were centrifuged (1000× *g* for 10 min at room temperature) within 2 h of venipuncture to separate plasma. Plasma samples were immediately frozen at −30 °C, and transferred to −80 °C within 1 week until analysis.

##### Sample Extraction

Plasma extraction was performed using a ternary methanol–water–chloroform extraction based on the established Folch and Bligh–Dyer procedures [24,25]. This liquid–liquid extraction separates polar, hydrophilic metabolites, including nucleobases, nucleosides, and nucleotides, into the upper aqueous/methanolic phase, while lipophilic compounds are retained in the lower chloroform phase.

A watery internal standard solution (4 µM of each compound) was prepared (adenine-1,3-15N2, adenosine-15-N65′-monophosphate, cytosine-2,4-13C215N3, uridine-5,6-d2). Twenty-five µL of the standard solution, 75 µL of methanol, and 75 µL of chloroform were added to 50 µL of sample volume, stirred, and the resulting ternary mixture was kept on ice for 15 min. If the sample volume was less than 50 µL, the remaining volume was filled with H_2_O. In order to separate the phases, the mixture was centrifuged at 3000× *g* and 4 °C for 20 min, resulting in an upper phase containing pyrimidines and purines and their derivatives and a lower phase that contained lipids. The upper phase was stored at −80 °C.

#### 2.3.2. Measured Purines, Pyrimidines, and Their Derivates

An overview of measured purines, pyrimidines, and their derivatives (nucleobases, nucleosides, and nucleotides) is given in Table 1.

#### 2.3.3. Analysis of Plasma Samples

Electrospray ionization tandem mass spectrometry (ESI MS/MS) was used to analyze purines and pyrimidines. Equipment included a Finnigan Surveyor Autosampler Plus, a Finnigan Surveyor MS Pump Plus, and a TSQ Quantum Ultra equipped with a heated electrospray ionization interface (H-ESI) (Thermo Fisher Scientific, Dreieich, Germany).

Pyrimidine and purine concentrations of the samples were determined in relation to those of the stable isotope-labelled internal standards and external calibration curves for all individual compounds. Linearity was assured for all compounds from the limit of detection onwards (Table 1). Limits of detection (LOD) were determined by serial dilution of a mixture containing all analytes at defined concentrations and were defined as the lowest concentration at which the respective analytes could be reliably detected under the applied analytical conditions. Adenine-1,3-15N2, uridine-5,6-d2-, and adenosine-15-N65’-monophosphate were used as internal standards for nucleobases, nucleosides, and nucleotide monophosphate, respectively. Purine and pyrimidine bases and their nucleosides were separated isocratically on a Zorbax SB-Aq RRHD column (2.1 × 150 mm inner diameter; 1.8 µm particle size) (Agilent Technologies, Waldbronn, Germany) at 50 °C with 0.1% formic acid in H_2_O. Nucleotide (nucleoside monophosphate) separation was performed on a Luna ^®^ Omega SUGAR 100 Å column (2.1 × 100 mm; 3.5 µm particle size, Phenomenex LTD, Aschaffenburg, Germany) at 35 °C via gradient elution using 0.1% formic acid in water (A) and 0.1% formic acid in acetonitrile (B). Analytical consistency across runs was monitored using aliquots of pooled control plasma stored at −80 °C and analyzed alongside study samples.

### 2.4. Statistical Analysis

We are not aware of any published data on purine and pyrimidine concentrations in cord blood of preterm or term neonates on which a power analysis could have been based. Gestational age was categorized into five predefined groups for statistical comparison (see Section 2.1), with the aim of including approximately 30 patients per group. With 30 samples per gestational age group, concentration differences between groups can be detected (in the case of normally distributed data) in the order of 0.75–1.0 standard deviations with an alpha of 0.05 and a power of 80% for 2-sided testing. Statistical analyses were performed using the statistical software JMP (SAS Institute Inc., Cary, NC, USA, Version 16). Data are shown as median (p25–p75) or mean (+/− 1 SD) if normally distributed. To assess correlations, Spearman’s correlation coefficient ρ was reported.

Analyte concentrations were analyzed using linear mixed-effects models, with group, sampling time point, and their interaction as fixed effects. Mother ID and infant ID were included as a random effect to account for multiple births and repeated measurements within the same infant, respectively. Independent residual errors were assumed. Values below the limit of detection (LOD) were set to zero. Group differences were first assessed, including all five groups. Longitudinal analyses were subsequently performed for the preterm groups, excluding term-born infants, because no measurements were available at d14 for the term-born infants. Significant fixed effects were followed by pairwise comparisons of least-squares means with Tukey–Kramer adjustment. Statistical significance was set at *p* < 0.05. For adenosine and GMP, a high proportion of measurements were below the LOD (0.01 µmol/L). Therefore, concentrations were converted into binary variables (detectable/non-detectable) and analyzed using generalized linear mixed-effects models with a binomial distribution and logit link. The same two-step approach regarding preterm and term-born infants and the longitudinal analysis was applied. In a secondary analysis, the linear mixed effects models were additionally adjusted for sex and small-for-gestational-age (SGA) status by including both variables as fixed effects. Paired variables were tested via the Wilcoxon test.

## 3. Results

### 3.1. Demographics

During the study period, 7566 infants were born at Tübingen University hospital; of these, parents of 222 infants in whom the study team was able to collect cord samples were approached and informed about the study, and after obtaining informed consent, one patient was subsequently excluded because of congenital malformations unknown at the time of inclusion. In total, 155 infant–mother pairs were included and available for statistical analysis (approval rate: 70.3%) (Figure 1). Since no needle sticks were permitted solely for study purposes, and since some patients had already been discharged by the time of the follow-up blood draw (day 3 and day 14), the number of day 14 samples decreased with increasing gestational age. In particular, among late preterm and term infants, few or no day 14 samples were collected, resulting in a knowledge gap attributable to the study design.

Table 2 provides an overview of the demographics of the different study groups. Demographic data of the eight additional pregnant women are shown in Table 3.

### 3.2. Nutrition

All participants were fed according to the standardized feeding guidelines of the Department of Neonatology, Tübingen, providing a rapid increase in enteral intake and initial partial parenteral nutrition. Enteral intake was about 50–60 mL/kg/d the day prior to the first postnatal blood sampling in all gestational groups. Preterm infants below 28 weeks’ gestation received donor milk instead of formula, if mother’s own milk supply was insufficient and parents consented. Therefore, the most immature infants of the study group received breast milk the day before blood sample 1 was taken, while the other, more mature infants were predominantly formula fed. Parenteral protein intake decreased with increasing gestational age, and fat infusion was only introduced in infants born before 32 weeks or at <1500 g birth weight. Since all mothers were encouraged and supported to express breast milk, the predominant source of enteral feeding 5 days prior to blood sampling 2 was the mothers’ own breast milk for preterm infants from 23 6/7 to 34 6/7 weeks. Only five children received a small proportion of parenteral nutrients prior to blood sample 2, while all others were receiving full enteral nutrition. Since there was very little data available for the gestational age group 35 0/7–36 6/7, the results should be interpreted with caution. Unfortunately, oral intake is not documented in detail on the general maternity ward, so no valid data from oral intake of the term infants was available. A detailed overview of the nutritional intake is shown in Table S1.

### 3.3. Blood Samples

Of the 19 target purines, pyrimidines, and their derivates (see Table 1), 11 could not be detected with the applied ESI-MS/MS method. In the following, only the analytes that were detected in at least one type of sample (cord blood, maternal, preterm infants’ blood), one gestational age group, and ≥50% of the samples are described. These were cytidine, uridine, hypoxanthine, xanthine, inosine, adenosine, AMP, and GMP. The percentage of values above the detection limit for each of these 8 metabolites is listed in Table S2 in the online supplement.

#### 3.3.1. Correlation of Pyrimidine and Purine Concentrations of Cord Blood with Gestational Age at Birth

To study the intrauterine plasma concentration of a fetus, umbilical cord plasma was analyzed, and correlation with gestational age at birth was tested. All analyzed purines, pyrimidines, and their derivates, except xanthine, GMP, and adenosine, showed a statistically significant negative correlation with gestational age at birth (Figure 2). Adenosine just missed the significance level with a *p*-value of 0.06. Admittedly, as the concentrations of adenosine, inosine, and GMP were only detectable in a minority of measured samples, correlations were only assessable to a limited extent. *p*-values and Spearman’s correlation coefficient (ρ) are shown in Figure 2. Linear regression analysis was performed to illustrate the relationship graphically.

#### 3.3.2. Pyrimidine and Purine Concentrations of Pregnant Women and Mothers Within 48 h of Birth

Significant differences between plasma concentrations of pregnant women and mothers who had already given birth were only shown for hypoxanthine and inosine (Table 4). However, concentrations of hypoxanthine and inosine in umbilical cord blood (detailed under Section 3.3.4 and Section 3.3.5) were significantly higher than in the blood of pregnant women or 48 h after birth.

#### 3.3.3. Pyrimidine Nucleosides: Cytidine and Uridine

Median plasma concentrations for cytidine and uridine are shown in Figure 3, and detailed information is listed in Table S3 (online supplement). Cytidine concentrations in maternal plasma were lower than in CB (*p* = 0.0001). Cytidine concentrations in cord plasma differed significantly between gestational age groups (*p* < 0.0001), with the highest concentrations observed in very immature preterm infants. In the mixed-effect model, cytidine concentrations differed significantly across gestational age groups (F (4, 138.0 = 13.96; *p* < 0.0001) and sampling time points (F (2, 249.0) = 41.14, *p* < 0.0001). Longitudinal analysis of the preterm groups showed a significant effect of time (F (2, 216.9) = 28.18, *p* < 0.0001) and a significant sampling time-by-gestational age group interaction (F = 4.51, *p* = 0.0003). In infants born at 23 0/7–27 6/7 wks, cytidine decreased from cord blood to day 3 and day 14 (estimated difference: 0.59µmol/L, 95% CI 0.26–0.91, and 0.82 µmol/L, 95% CI 0.48–1.16; both Tukey-adjusted *p* < 0.0001), with no further significant change between days 3 and 14 (*p* = 0.52). In preterm infants born at 28 0/7–31 6/7 wks, concentrations were significantly lower on day 3 (estimated difference: 0.38 µmol/L, 95% CI 0.01–0.64) and on day 14 (estimated difference: 0.58, 95% CI 0.25–0.90, *p* < 0.0001) than in cord blood, whereas no significant longitudinal changes were observed in infants > 31 6/7 weeks.

As shown for cytidine, uridine concentrations in maternal plasma were lower than in CB (*p* = 0.0001). Uridine concentrations in cord plasma differed significantly across gestational age groups (F(4, 99.5) = 10.2, *p* < 0.0001). Pairwise comparisons revealed a transient increase in uridine concentrations from cord blood to day 3 (estimated difference: 1.4 µmol/L, 95% CI 0.64–2.23, Tukey–Kramer adjusted *p* < 0.0001), followed by a decrease from day 3 to 14 (estimated difference: 1.44, 95% CI 0.40–2.49, *p* = 0.0035), while concentrations in cord blood and at day 14 did not differ (*p* = 0.9995). In the longitudinal analysis, significant effects of sampling time (F(2, 230.6) = 6.37, *p* = 0.002) and gestational age group (F(3, 72.9) = 5.18, *p* = 0.0027) were observed, whereas the sampling time-by-gestational age group interaction was not significant (F(6, 225.4) = 0.51, *p* = 0.80), providing no evidence of a differential postnatal course across gestational age groups.

#### 3.3.4. Purine Nucleobases: Hypoxanthine and Xanthine

Plasma concentrations for hypoxanthine and xanthine are shown in Figure 3, with details listed in Table S4 (median; p25–p75). Lowest hypoxanthine concentrations were found in the plasma of mothers. Hypoxanthine concentrations decreased significantly from cord blood to day 3 (estimated difference: 5.48 µmol/L, 95% CI: 3.06–7.89) and further to day 14 (estimated difference: 6.06 µmol/L, 95% CI: 2.91–9.21; both Tukey-adjusted *p* < 0.0001). In the longitudinal mixed-effects model, concentrations differed significantly between time points (F(2, 231.5) = 28.39, *p* < 0.0001) and between gestational age groups (F(3, 88.1) = 5.61, *p* = 0.0014). There was no significant interaction between time point and gestational age group (F(6, 223.1) = 1.33, *p* = 0.246), indicating a comparable temporal pattern across gestational age groups.

Lowest xanthine concentrations were found in maternal plasma. Xanthine concentrations decreased from cord blood to postnatal day 3 (estimated difference: 0.19 µmol/L, 95% CI: 0.02–0.37, Tukey-adjusted *p* = 0.028) and further to day 14 (estimated difference: 0.31 µmol/L; 95% CI: 0.08–0.54, *p* = 0.0050). In the longitudinal mixed-effects model (only preterm infants), xanthine concentrations differed significantly between time points (F(2, 223.9) = 7.98, *p* = 0.0004), whereas neither gestational age group (F(3, 86.9) = 1.59, *p* = 0.199) nor the interaction between time point and gestational age group were significant (F(6, 215.2) = 0.75, *p* = 0.608).

#### 3.3.5. Purine Nucleosides: Adenosine and Inosine

The median adenosine concentration in plasma of mothers or in CB was below the detection limit, and only very low concentrations were shown in the plasma of infants studied on d3. Adenosine detectability differed significantly by time point (F(2, 363) = 12.05, *p* < 0.0001) and gestational age group (F(4, 109.1) = 5.37, *p* = 0.0006). Adenosine was more likely to be detectable on d3 than in cord blood (Tukey-adjusted *p* < 0.0001) or on d14 (*p* = 0.0464). No significant time-by-gestational age group interaction (F (6, 300) = 0.79, *p* = 0.579) was observed.

Inosine concentrations in maternal plasma were below the limit of detection in all gestational age groups except for mothers of term infants, and the highest concentrations were observed in the cord plasma of very preterm infants. Among preterm infants, inosine concentrations did not change significantly over time (F(2, 236.8) = 2.23, *p* = 0.11). Neither the gestational age group (F(3, 118.8) = 1.95, *p* = 0.126) nor the interaction between time point and gestational age group (F(6, 197.6) = 1.74, *p* = 0.114) was significant. At postnatal day 14, inosine concentrations were rarely above LOD; see Figure 3. Detailed adenosine and inosine concentrations (median; p25–p75) are listed in Table S5.

#### 3.3.6. Purine Nucleotides: Adenosine (AMP) and Guanosine Monophosphate (GMP)

AMP concentrations differed significantly by time point (F(2, 272.4) = 6.37, *p* = 0.002) and gestational age group (F(4, 79.2) = 4.91, *p* = 0.0014) in the main mixed-effects model. AMP concentrations were significantly higher on postnatal day 3 than in cord blood (estimated difference: 1.28 µmol/L, 95% CI: 0.34–2.22, Tukey-adjusted *p* = 0.0041) and on postnatal day 14 (estimated difference: 1.38, 95% CI: 0.15–2.60, *p* = 0.023), whereas cord blood and day 14 did not differ (*p* = 0.981). In the longitudinal analyses restricted to preterm infants, the time-by-gestational age group interaction showed statistical significance (F(6, 229.5) = 2.48, *p* = 0.024).

GMP detectability differed significantly over time (*p* = 0.0004), but not between gestational age groups (*p* = 0.081). There was a significant difference of detectability between cord blood and d3 (Tukey adjusted *p* = 0.0002). Among preterm infants, no significant time-by-group interaction was observed (*p* = 0.649). The detailed AMP and GMP concentrations (median; p25–p75) are listed in Table S6.

#### 3.3.7. Adjusted Mixed Model/GLMM

All models were additionally adjusted for sex and SGA status. Overall, adjustments did not substantially alter the main findings. However, SGA status showed a significant independent effect on uridine concentrations (*p* = 0.0005). For most metabolites, previously observed time and group effects remained unchanged. For AMP, the previously significant group-by-time interaction (*p* = 0.024) was attenuated after adjustment and was not longer statistically significant (*p* = 0.053). Thus, adjustment for sex and SGA status largely confirmed the primary findings, with relevant changes confined mainly to effects observed for AMP.

## 4. Discussion

The fetus undergoes a growth spurt between the 24th and the 34th week of gestation, with 3–4-fold higher growth rates compared to term infants during the first postnatal month, resulting in increased nutritional needs. Correspondingly, this study found particularly high purine and pyrimidine concentrations in fetuses with a low gestational age (GA). These compounds have various functions in the human organism, such as genetic coding (DNA), protein synthesis (RNA), and intermediate metabolism (CTP for phospholipids, UTP and GTP for carbohydrates, adenosine for methyl transfer). Increased cord blood concentrations rapidly decreasing after birth could indicate changes in endogenous synthesis, cellular uptake, and metabolism during a preterm infant’s adaptation to the environment, but also an active placental transfer to meet increased requirements and turnover in fast-growing organisms with higher needs to ensure adequate growth followed by reduced postnatal supply. Notably, while the pathways for nucleobase synthesis are generally present, requirements may exceed synthesis capacity during rapid growth, as shown for other conditionally essential nutrients in the fetus and (preterm) infant [26,27] (see below). Recent fecal metabolomic data provide additional evidence for gestational age-related differences in pyrimidine metabolism, with particularly pronounced alterations reported in infants before 32 weeks of gestation [28]. Although these findings cannot be directly extrapolated to our findings, they support the concept that pyrimidine metabolism may vary with developmental maturity.

Mothers and parturients in this study had significantly lower plasma concentrations of purines and pyrimidines than their fetuses, regardless of the week of gestation. Whereas most maternal samples were very early postpartum samples, these likely reflect pre-partum conditions because we did not observe differences in plasma concentrations in pregnant women (Table 3). This observation may have several explanations, including differences in fetal cellular uptake or metabolism, as well as potential placental transfer against a concentration gradient to meet the fetus’ increased requirements. While the latter mechanism cannot be proven from the present data, active and selective placental transfer against a concentration gradient has been demonstrated for selected purine metabolites in experimental guinea pig models [29] and other essential nutrients, including choline, arachidonic acid, and docosahexaenoic acid [26,27]. Whether (some) purines and pyrimidines are subject to similar mechanisms to support the growth of the rapidly growing fetus remains to be determined. Recent metabolomic data further suggests that placental purine metabolism is altered in preterm birth, with differences in several purine metabolites reported between preterm and term placentas [30]. Although these findings do not provide evidence for active placental transport, they support the potential involvement of placental nucleotide metabolism during the third trimester.

Further evidence of the important role of pyrimidines and purines in children’s growth comes from findings showing that supplementation with nucleotides (CMP, AMP, GMP, UMP, IMP) leads to catch-up growth in SGA newborns [11]. This finding provides further biological rationale for investigating the potential role of purines and pyrimidines in supporting growth, although its relevance to preterm infants and to the compounds measured in the present study remains uncertain.

In this study, only the nucleobases xanthine and hypoxanthine, the nucleosides cytidine, uridine, inosine, and adenosine, and the nucleotides AMP and GMP were detected in measurable concentrations, while other compounds were below the detection level. Among the quantifiable compounds, however, there were significant differences in their plasma concentrations, depending on gestational age at birth and source (cord blood, postnatal, or maternal plasma). Xanthine, hypoxanthine, inosine, and cytidine showed a drastic fall in plasma concentrations within 3 and 14 days of birth, while uridine remained high during this period. These postnatal differences remained visible across comparable PMAs; however, as these comparisons are descriptive, developmental maturation cannot be excluded as a contributing factor (Supplemental Figure S1a,b). Directly comparable reference data are scarce; however, in a recent study, concentrations in term pregnancies of hypoxanthine and xanthine (maternal 3.87–4.85 µmol/L; cord 10.6–13.9 µmol/L for hypoxanthine, and maternal 0.79–1.00 µmol/L; cord 1.05–1.45 µmol/L for xanthine) were reported [31]. Hypoxanthine concentrations were broadly consistent with our findings, whereas xanthine concentrations were at least one order of magnitude higher than in the present study. Comparable data for the other metabolites are lacking.

### 4.1. Purines

The interpretation of purine concentrations is complex, as detectable purines and purine derivatives are largely involved in energy metabolism as well as cellular communication and second messenger systems. All investigated groups show remarkable differences in this context, suggesting that differences in concentrations may largely reflect metabolic rather than nutritional causes.

The nucleotides GMP and AMP, as well as the nucleoside adenosine, were found in higher concentrations in the postnatal blood samples on d3 and d14 than in the fetus or mother. Xanthine and hypoxanthine are degradation products of GMP and AMP (and adenosine and inosine) and are metabolized to uric acid as their end product of degradation. In the case of a hypoxic environment (such as childbirth, especially complicated childbirth), AMP is degraded to hypoxanthine to obtain a high energy charge [32], and xanthine oxidase (hypoxanthine → xanthine) is inhibited [33]. Prolonged hypoxia is therefore associated with increased hypoxanthine levels, and hypoxanthine has been studied in the past as a surrogate marker for hypoxia/ischemia. Our study showed increased hypoxanthine levels (up to 71.3 µmol/L) in the umbilical cord, which, however, showed no correlation with cord blood pH (as an indicator of hypoxia) of the measured samples. Nonetheless, the increased hypoxanthine and xanthine levels (and their decrease within 14d of birth) could partly be due to the environmental conditions and represent the change from the physiologically hypoxic intrauterine compartment to the extrauterine environment with higher partial pressures of oxygen. Hence, these changes could primarily be a sign of a changing metabolism, oxygen pressure, and the energy needs of the infant.

Adenosine concentrations were only above the LOD in the samples measured on d3. In addition to its role as a component of DNA and RNA, adenosine acts as a regulator in many metabolic processes, including neuronal plasticity [34] and memory and learning [35,36], and is, in the form of ATP, the body’s ubiquitous cellular energy source [3]. Concentrations of adenosine are strictly regulated, but a slight increase in plasma concentrations occurs when intracellular ATP is degraded. However, this degradation leads to increased plasma concentrations of inosine and hypoxanthine rather than adenosine, in keeping with a rapid turnover [37].

Inosine is an intermediate product of the degradation of adenosine to hypoxanthine, and its role in metabolic processes, probably (amongst others) as a regulator of immunity, is poorly understood [38]. Notably, there were especially high fetal plasma concentrations of inosine before 28 weeks of gestation and a drop in preterm infants’ levels within 14 days of birth. In line with the relationship of hypoxanthine and inosine metabolism, the concentrations of hypoxanthine and inosine followed a similar time course. The high concentrations directly after birth may be the result of increased energy requirements of preterm infants during the transitional phase (with extraordinary stress during adaptation from intrauterine to extrauterine). The following decrease up to day 14 may reflect both (1) that the situation (in general and in terms of energy requirements) of the preterm infant has stabilized during the first two weeks after birth and (2) that the postnatal increase in tissue oxygen tension facilitates oxidative phosphorylation. Notably, when interpreting the context of inosine/hypoxanthine and adenosine concentrations and the organism’s varying energy requirements, the findings in preterm infants are consistent with those in adult study participants (pre- and post-partial women). The only detectable differences between pregnant women and parturients were their hypoxanthine and inosine concentrations. In line with this, pregnant women have a higher energy requirement, especially in the second and third trimesters [39], and the levels of hypoxanthine and inosine of the fast-growing fetus were much higher than in the observed adults throughout.

### 4.2. Pyrimidines

While fluctuations in purine concentrations appear to be primarily attributable to energy metabolism, the dietary context may play a greater role in the case of pyrimidines.

The pyrimidine nucleosides cytidine and uridine were detected in relevant concentrations in all samples. While uridine concentrations showed a transient increase at day 3 but returned to cord blood levels by day 14, those of cytidine fell continuously until d14. Uridine is necessary for RNA and glycogen synthesis and for phospholipid synthesis after conversion to cytidine, e.g., for cell membrane formation during parenchymal growth [40]. Plasma concentrations of uridine are shown to be the highest of all pyrimidine nucleosides and bases [41]. Since pyrimidines are recycled from their nucleosides (and not from their bases like purines), the constantly high uridine concentration indicates that uridine is the main source for the synthesis of all pyrimidine nucleotides [41]. Interestingly, recent longitudinal data from extremely preterm infants identified an inverse association between serum uridine concentrations and enteral energy intake during the first 28 postnatal days [42], indicating that nutrition should be considered as a potential factor influencing circulating uridine concentrations. However, despite stable levels of uridine, the decreasing concentrations of cytidine, which is required for DNA synthesis and phospholipid synthesis, may indicate its decreased availability for cell proliferation and may contribute to the decreased parenchymal growth of preterm infants. Notably, thymidine synthesis requires a one-carbon transfer, depending on tetrahydrofolate that is fed by one-carbon donors like choline [43].

Compared to plasma concentrations of adults (males 4.9 ± 0.7 µmol/L; non-pregnant females 4.5 ± 0.7 µmol/L [44]), mothers in this study had lower uridine plasma concentrations, whereas those in preterm fetuses and infants were higher. These higher concentrations may indicate active placental transport towards the fetus. Concentration differences were particularly high below 32 weeks of gestation, i. e., during the growth spurt of parenchymal organs. Higher concentrations, particularly of pyrimidines for intermediate metabolism and formation of structural phospholipids, may result in greater availability for parenchymal growth. Hence, enteral supplementation represents a promising approach for future studies aimed at demonstrating a potential effect on the outcomes of preterm infants.

### 4.3. Preterm Infants’ Supply with Purines and Pyrimidines

At present, the nutritional needs of preterm infants regarding purines and pyrimidines are unclear. In general, a pyrimidine-rich diet has the potential to increase plasma levels (in contrast to a purine-rich diet, where purines are primarily metabolized directly to uric acid) [45,46,47]. Breast milk content of purines and pyrimidines varies considerably. However, the pyrimidine nucleosides cytidine and uridine, as well as the pyrimidine nucleotides CMP and UMP, are predominant [48,49]. The nucleotide profile appears to be partly due to the enzymatic degradation of the RNA contained in breast milk, as the milk contains the corresponding enzymes and the end product uric acid from purines. The predominant nucleosides cytidine and uridine are produced in this process [49]. It is possible that additional supplementation with pyrimidines (and purines?) leads to increased plasma concentrations of these nutrients and, in turn, may contribute to improved parenchymal growth in preterm infants. However, this is the subject of further clinical investigation.

Our finding should be interpreted with caution. First, as this study was designed as a pilot study, the sample size within each group was relatively small. Nevertheless, the sample size allowed for the detection of some relevant between-group differences. Second, due to the observational nature of this study, reported associations cannot be interpreted as causal relationships—derived hypotheses have thus to be tested in future interventional studies. Furthermore, concentrations of numerous analytes were below the lower limit of detection (LOD) in substantial proportions of samples. Pyrimidines and purines below the respective LOD could not be reliably quantified and, hence, cannot be assessed within the scope of this study. For analytes analyzed continuously, assigning measurements below the LOD to zero may have influenced the estimated effects and should be considered when interpreting the results. In addition, the analytical method was not specifically validated for the present sample matrices; however, stable isotope-labeled internal standards were used to account for potential matrix effects. Moreover, maternal nutritional status and hence maternal and fetal blood and human milk concentrations of purines and pyrimidines may be influenced by cultural background, dietary habits, and regional nutritional practices beyond the crude dietary restrictions reported herein. As these factors may differ across populations, the present findings may not be directly generalizable to populations with different demographic or cultural characteristics. In addition, as the nutritional mode of the infants was strongly associated with gestational age and postnatal age and showed limited overlap across the five gestational age groups, its independent contribution to differences in metabolite concentrations could not be reliably assessed.

In summary, the plasma concentration of uridine in premature infants’ plasma is constantly higher than adult levels. Cytidine concentration was only increased in cord blood and decreased rapidly after birth. In the above context of the interconnected metabolic pathways of uridine and cytidine, their contrasting concentration profiles may provide a hypothesis for further studies to determine whether pyrimidine requirements (especially cytidine) are increased during the fetal/preterm infant growth spurt, i. e., before 34 weeks’ gestation. However, as the relationships between purine and pyrimidine concentrations and their metabolism are extremely complex, further studies are needed to better understand their impact on the nutritional needs of preterm infants.

## 5. Conclusions

Concentrations of several purines and pyrimidines and their derivatives were higher in fetal than in maternal plasma, demonstrating marked maternal-fetal concentration differences. Postnatally, particularly in preterm infants, concentrations of several metabolites, including cytidine, inosine, hypoxanthine, and xanthine, decreased markedly. The mechanisms underlying these differences cannot be determined from the present observational data and may include placental transport, endogenous synthesis, tissue uptake, metabolism, and nutritional intake. Whether nutritional supplementation with purines and pyrimidines could influence plasma concentrations or growth and development in preterm infants remains to be investigated in future studies.

## Figures and Tables

**Figure 1:**
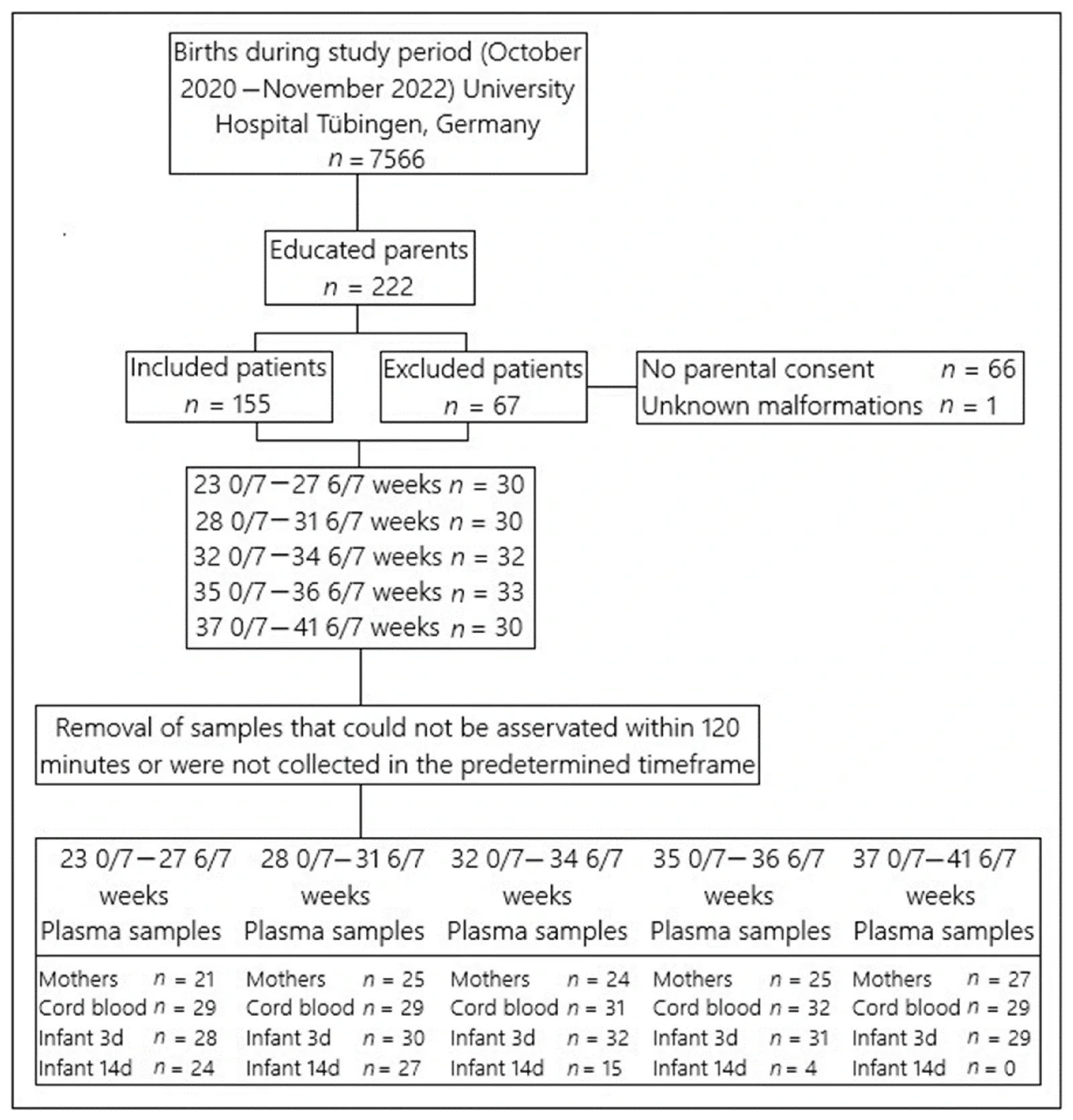
CONSORT flowchart of the study population.

**Figure 2:**
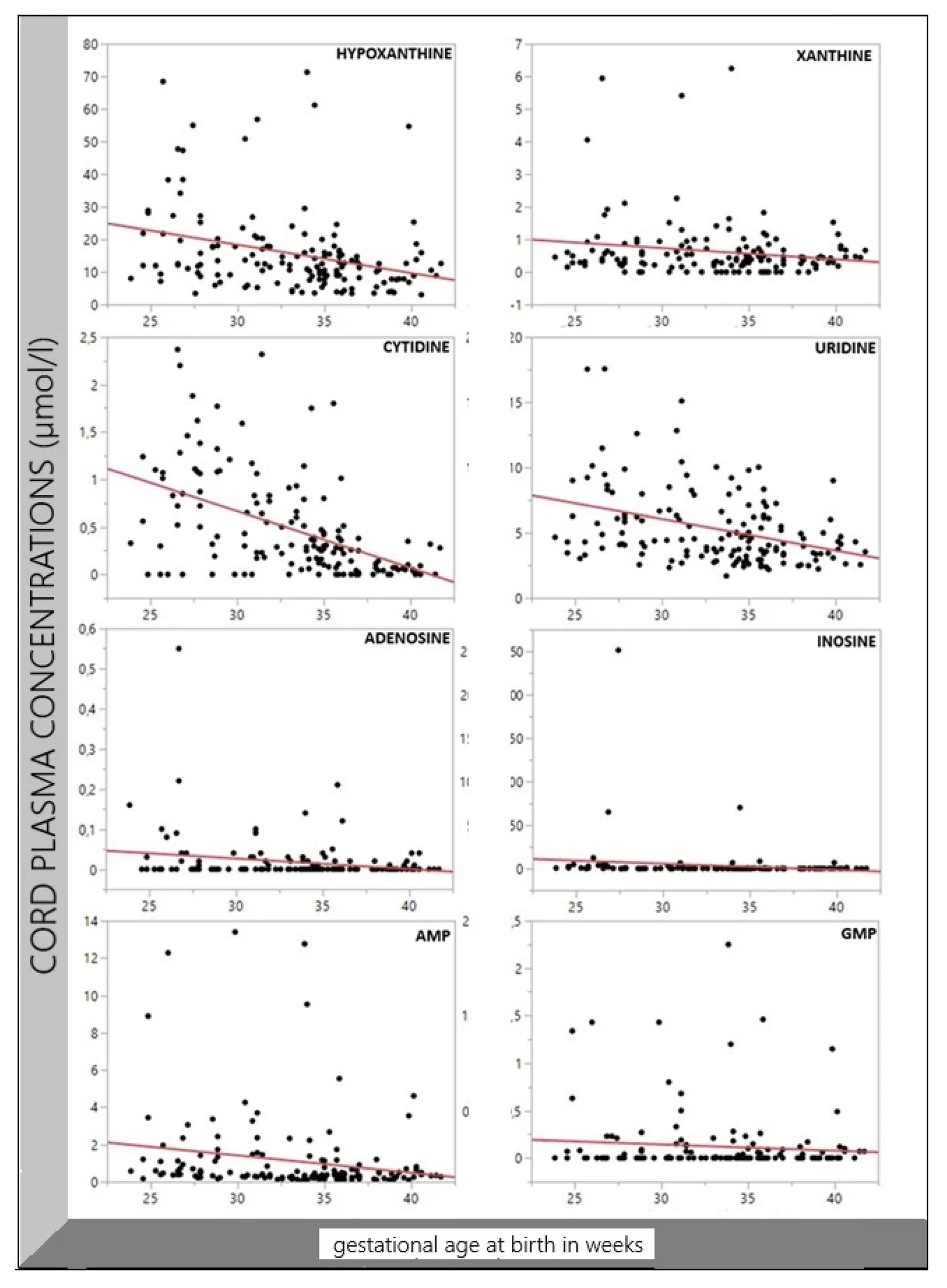
Graphs indicating individual cord plasma concentrations in µmol/L versus gestational age at birth in weeks. *p*-values and Spearman correlation coefficient ρ: hypoxanthine *p* < 0.001; ρ = −0.33; xanthine *p* = 0.12; ρ = −0.13; cytidine *p* < 0.0001; ρ = −0.51; uridine *p* < 0.0001, ρ = −0.36; inosine *p* < 0.0001, ρ = −0.43; adenosine *p* = 0.06, ρ = −0.15; AMP *p* = 0.0007, ρ = −0.28; GMP *p* = 0.8, ρ = −0.02; Abbreviations: AMP, adenosine monophosphate; GMP, guanosine monophosphate.

**Figure 3:**
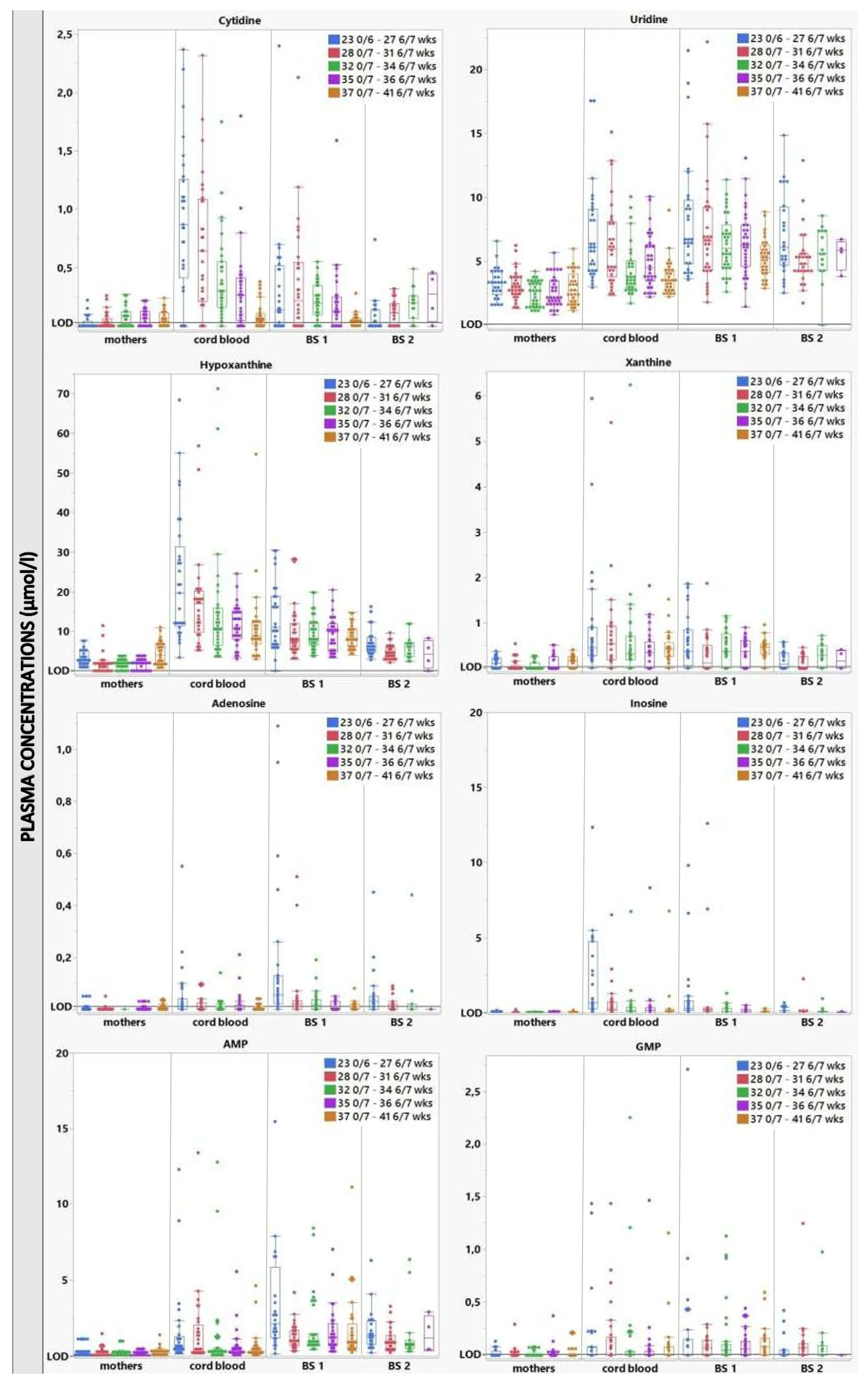
Boxplots and individual dots of pyrimidine and purine plasma concentrations (µmol/L) in mothers, cord plasma, and plasma of infants at BS 1 (3 days) and BS 2 (14 days) by gestational age group. Numbers of available observations for each gestational age group: 23 0/6–27 6/7 wks: mothers: n = 21, cord blood: n = 29, BS1 n = 28, BS2 n = 24; 28 0/7–31 6/7 wks: mothers: n = 25, cord blood: n = 29, BS1 n = 30, BS2 n = 27; 32 0/7–34 6/7 wks: mothers: n = 24, cord blood: n = 31, BS1 n = 32, BS2 n = 15; 35 0/7–36 6/7 wks: mothers: n = 25, cord blood: n = 32, BS1 n = 31, BS2 n = 4; 37 0/7–41 6/7 wks: mothers: n = 27, cord blood: n = 29, BS1 n = 29, BS2 n = 0. For improved visualization, the y-axis of AMP and inosine was limited to 20 µmol/L. Three observations each (all measured in cord blood) exceeded this range (but were included in the statistical analysis). Abbreviation: adenosine monophosphate (AMP); guanosine monophosphate (GMP); limits of detection (LOD); blood sample (BS); weeks (wks).

**Table 1:** Measured purines, pyrimidines, and their derivatives with limits of detection (LOD).

Pyrimidine–Nucleobase	Nucleoside	Nucleotide Monophosphate
Cytosine (0.1 µmol/L)	Cytidine (0.03 µmol/L) ^X^	CMP (0.01 µmol/L)
Thymine (0.03 µmol/L)	Thymidine (0.01 µmol/L)	TMP (1.0 µmol/L)
Uracil (1.0 µmol/L)	Uridine (0.1 µmol/L) ^X^	UMP (3.0 µmol/L)
**Purine–Nucleobase**	**Nucleoside**	**Nucleotide monophosphate**
Adenine (0.03 µmol/L)	Adenosine (0.01 µmol/L) ^X^	AMP (0.01 µmol/L) ^X^
Guanine (0.03 µmol/L)	Guanosine (0.01 µmol/L)	GMP (0.01µmol/L) ^X^
Hypoxanthine (0.1 µmol/L) ^X^	Inosine (0.01 µmol/L) ^X^	IMP (0.3 µmol/L)
Xanthine (0.03 µmol/L) ^X^		

Abbreviations: AMP, adenosine monophosphate; CMP, cytidine monophosphate; GMP, guanosine monophosphate; IMP, inosine monophosphate; TMP, thymidine monophosphate; UMP, uridine monophosphate. ^X^ indicates the nutrients measured above LOD in this study.

**Table 2:** Demographic data of infants and their mothers according to gestational age group, including the number of small-for-gestational-age infants (birthweight lower 10th percentile).

	Gestational Age Group (Weeks)				
	23 0/7–27 6/7	28 0/7–31 6/7	32 0/7–34 6/7	35 0/7–36 6/7	37 0/7–41 6/7
*N*	30	30	32	33	30
Sex (m/f) *n*/*n*	14/16	23/7	20/12	14/19	18/12
Birthweight	761 (±182)	1390 (±403)	2094 (±465)	2494 (±456)	3328 (±664)
Singletons *n* (%)	17 (57%)	23 (77%)	15 (47%)	20 (61%)	26 (87%)
Maternal age (years)	32.6 (±4.6)	31.7 (±3.4)	34.6 (±5.1)	34.0 (±3.9)	31.6 (±4.6)
Maternal diet, *n* (%)No restrictions No fishVegetarianVeganMissing data	20 (91%)1 (5%)1 (5%)08	18 (78%)2 (9%)2 (9%)1 (4%)7	23 (100%)0009	18 (86%)2 (10%)1 (5%)012	21 (95%)1 (5%)008
Small for gestational age *n* (%)	10 (33%)	7 (23%)	6 (19%)	6 (18%)	7 (23%)

Data are the mean (± SD) or *n* (%) of the indicated numbers (*N*) of individuals.

**Table 3:** Demographic data of the additional pregnant women.

	*N*	Maternal Age (Years)	Week of Gestation When Blood Sample Was Drawn	Singletons
Pregnant woman	8	29.5 (29.0–34.5)	30.3 (27.4–32.8)	6/8

Data are the medians (p25–p75) of the indicated number (*N*) of individuals.

**Table 4:** Overview of the purine and pyrimidine plasma concentrations of pregnant women and women after birth.

		Pregnant Women	Women After Birth	
	LOD (µmol/L)	Plasma Concentrations in µmol/L; Median (p25–p75)		*p*
Cytidine	0.03	0.03 (<0.03–0.04)	<0.03 (<0.03–0.10)	n.s.
Uridine	0.1	3.19 (2.70–3.40)	2.78 (1.88–3.65)	n.s.
Hypoxanthine *	0.1	4.49 (2.86–5.90)	1.86 (1.14–3.76)	0.01
Xanthine	0.03	<0.03 (<0.03–0.19)	<0.03 (<0.03–0.20)	n.s.
Adenosine	0.01	<0.01 (<0.01–0.01)	<0.01 (<0.01–<0.01)	n.s
Inosine *	0.01	0.05 (0.03–0.07)	<0.01 (<0.01–0.03)	0.0003
AMP	0.01	0.18 (0.08–0.32)	0.17 (0.10–0.27)	n.s.
GMP	0.01	<0.01 (<0.01–<0.01)	<0.01 (<0.010–<0.01)	n.s.

Abbreviations: limit of detection (LOD); adenosine monophosphate (AMP); guanosine monophosphate (GMP), * indicates statistical significance.

## Data Availability

Data are contained within the article.

## Supplementary Materials

The following supporting information can be downloaded at https://www.mdpi.com/article/10.3390/nu18203351/s1, Table S1: Overview of nutrient intake (enteral/parenteral) before blood samples 1 and 2. Data are presented as median (IQR) unless otherwise indicated. Predominant enteral feeding was defined as >75% of enteral intake from the respective source. For parenteral nutrients, n indicates the number of infants receiving the respective nutrient. Table S2: Percentage of measured concentrations of each metabolite above the limit of detection (LOD) in each sample group. Table S3: Plasma cytidine and uridine concentrations (µmol/L) in the mothers (M), cord blood (CB), and the infants at 3 days (3d) and 14 days (14d) for each study group. Table S4: Plasma hypoxanthine and xanthine concentrations (µmol/L) in the mothers (M), cord blood (CB), and the infants at 3 days (3d) and 14 days (14d) for each study group. Table S5: Plasma adenosine and inosine concentrations (µmol/L) in the mothers (M), cord blood (CB), and the infants at 3 days (3d) and 14 days (14d) for each study group. Table S6: Plasma adenosine-monophosphate (AMP) and guanosine-monophosphate (GMP) concentrations (µmol/L) in the mothers (M), cord blood (CB), and the infants at 3 days (3d) and 14 days (14d) for each study group. Figure S1a: Plasma concentrations (µmol/L) of cytidine, uridine, hypoxanthine, and xanthine according to postmenstrual age at blood sampling. Individual measurements are shown for cord blood, postnatal day 3, and postnatal day 14. Lines represent spline-smoothed median concentrations, with shaded areas indicating 95% confidence intervals. Colors and symbols distinguish the different sampling time points (blue = cord blood, red = postnatal day 3, green = postnatal day 14). Five observations (xanthine) are above the displayed y-axis. These observations were retained in the statistical analyses. Figure S1b: Plasma concentrations (µmol/L) of adenosine, inosine, AMP, and GMP according to postmenstrual age at blood sampling. Individual measurements are shown for cord blood, postnatal day 3, and postnatal day 14. Lines represent spline-smoothed median concentrations, with shaded areas indicating 95% confidence intervals. Colors and symbols distinguish the different sampling time points (blue = cord blood, red = postnatal day 3, green = postnatal day 14). Two observations (adenosine), three observations (inosine), one observation (AMP), and two observations (GMP) are above the displayed y-axis. These observations were retained in the statistical analyses.

## Abbreviations

The following abbreviations are used in this manuscript:

ADP	Adenosine diphosphate
AMP	Adenosine monophosphate
ATP	Adenosine triphosphate
C	Celsius
CB	Cord blood
CDP	Cytidine diphosphate
CMP	Cytidine monophosphate
CTP	Cytidine triphosphate
d	days
DNA	Deoxyribonucleic acid
EDTA	Ethylenediaminetetraacetic acid
ESI-MS/MS	Electrospray Ionization Tandem Mass Spectrometry
GA	Gestational age
GDP	Guanosine diphosphate
GMP	Guanosine monophosphate
HPLC	High-Performance Liquid Chromatography
IMP	Inosine monophosphate
LOD	Limit of Detection
n.s.	Not significant
NICU	Neonatal Intensive Care Unit
NPN	Non-protein nitrogen
RNA	Ribonucleic acid
SGA	Small for gestational age
UDP	Uridine diphosphate
UMP	Uridine monophosphate
