# Supplementary material for: Pyrimidine and Purine Concentrations in Cord, Preterm and Term Infant Plasma Compared to Maternal Values—An Observational Study
Source: Nutrients. 2026 Oct 13;18(20):3351. doi: 10.3390/nu18203351 (PMC13708128; doi:10.3390/nu18203351)
Supplement: Supplementary file 1 [file nutrients-18-03351-s001.zip › Table S1 - Table S6_FigureS1a_und_S1b_proofread.pdf]

Table S1

	23 6/7 – 27 6/7 weeks	28 0/7 – 31 6/7 weeks	32 0/7 – 34 6/7 weeks	35 0/7 – 36 6/7 weeks
	n = 30<br>missing data = 4	n =30<br>missing data = 0	n = 32<br>missing data = 0	n = 33<br>missing data = 7
Intake 1 day prior to blood sample 1				
Enteral feeding<br>(ml/kg/d)	53.1<br>(28.6 – 61.3)	54.5<br>(40.1 – 62.8)	60.4<br>(43.7 – 87.9)	57.1<br>(49.1 – 67)
Predominant intake (>75%)<br>MoM (n)<br>Donor milk (n)<br>Formula (n)<br>Mixed (n)	 4<br>18<br>3<br>1	 2<br>2<br>24<br>2	 0<br>0<br>29<br>3	 0<br>0<br>23<br>3
Parenteral protein<br>(n)<br>(g/kg/24h)	 26<br>3.47 (3.22 – 3.64)	 29<br>3.02 (2.41 – 3.38)	 16<br>2.09 (2.01 – 2.72)	 4<br>1.99 (1.56 – 2.25)
Parenteral fat<br>(n)<br>(g/kg/24h)	 26<br>2.01 (1.99-2.08)	 21<br>2.00 (2.00 – 2.02)	 3<br>2.02 (2.01 – 2.07)	 0
Mean daily intake during the 5 days prior to blood sample 2				
	n = 30<br>missing data = 3	n = 30<br>missing data =2	n = 32<br>Missing data = 17	n = 33<br>missing data = 30
Enteral feeding<br>(ml/kg/d)	141.5<br>(133.2 – 155.2)	149.8<br>(144.2 – 154.9)	142.3<br>(118.3 – 146.6)	142.4<br>(142.4 – 152.1)
MoM (>75%)<br>Breastmilk (n)<br>Donor milk (n)<br>Formula (n)<br>Mixed (n)	 20<br>1<br>0<br>6	 21<br>0<br>4<br>3	 10<br>0<br>3<br>2	 1<br>0<br>2<br>0
Parenteral protein<br>(n)<br>(g/kg/24h)	 4<br>1.35 (0.6 – 1.8)	 1<br>0.13	 0	 0
Parenteral fat<br>(n)<br>(g/kg/24h)	 3<br>0.74 (0.26 – 1.37)	 0	 0	 0

Table S1- Overview of nutrient intake (enteral/parenteral) before blood samples 1 and 2. Data are presented as median (IQR) unless otherwise indicated. Predominant enteral feeding was defined as >75% of enteral intake from the respective source. For parenteral nutrients, n indicate the number of infants receiving the respective nutrient, abbreviation: Mother´s own breastmilk (MoM)

Table S2

	Percentage of Measured Concentrations above Limit of Detection (LOD) (%)			
	Mother	Cord Blood	Postnatal 3d±1	Postanatal d14±2
Cytidine	49	82	70	70
Uridine	100	100	100	100
Xanthine	39	81	70	51
Hypoxanthine	79	100	99	99
Adenosine	16	31	58	49
Inosine	28	87	77	34
AMP	98	100	100	100
GMP	18	36	59	49

Table S2 – Percentage of measured concentrations of each metabolite above the limit of detection (LOD) in each sample group

Table S3

	Cytidine concentrations in µmol/l; median (p25-p75)									
	23 0/7 – 27 6/7w	n	28 0/7 – 31 6/7w	n	32 0/7 – 34 6/7w	n	35 0/7 – 36 6/7w	n	37 0/7 – 41 6/7w	n
M	<0.03 (<0.03 – 0.08)	21	<0.03 (<0.03 – 0.08)	25	0.03 (<0.03 – 0.11)	24	0.05 (<0.03 – 0.12)	25	0.04 (<0.03 – 0.11)	27
CB	0.86 (0.37 – 1.21)	29	0.64 (0.21 – 1.09)	29	0.30 (0.16 – 0.55)	31	0.27 (0.05 – 0.41)	32	0.05 (<0.03 – 0.11)	29
3d	0.15 (<0.03 – 0.53)	28	0.22 (<0.03 – 0.55)	30	0.20 (0.10 – 0.35)	32	0.13 (<0.03 – 0.25)	31	0.04 (<0.03 – 0.07)	29
14d	<0.03 (<0.03 – 0.14)	24	0.11 (<0.03 – 0.19)	27	0.20 (0.07 – 0.26)	15	0.28 (0.04 – 0.45)	4	-	0
	Uridine concentrations in µmol/l; median (p25-p75)									
M	3.30 (2.50 – 4.27)	21	3.11 (2.29 – 3.79)	25	2.54 (1.51 – 3.24)	24	2.04 (1.71 – 3.45)	25	2.77 (1.97 – 4.21)	27
CB	5.98 (3.79 – 8.11)	29	5.93 (3.79 – 8.11)	29	3.74 (2.89 – 5.02)	31	4.49 (3.44 – 6.30)	32	3.44 (2.67 – 4.33)	29
3d	6.70 (4.89 – 9.90)	28	6.56 (4.27 – 9.18)	30	6.05 (4.53 – 7.84)	32	6.27 (4.60 – 7.84)	31	5.27 (4.12 – 6.26)	29
14d	6.09 (4.70 – 9.23)	24	4.81 (4.19 – 5.62)	27	5.64 (4.26 – 7.35)	15	5.87 (4.32 – 6.54)	4	-	0

Table S 3 - Plasma cytidine and uridine concentrations (µmol/l) in the mothers (M), cord blood (CB), and the infants at 3 days (3d) and 14 days (14d) for each study group, shown as median (p25-p75), n= number samples analyzed

Table S4

	Hypoxanthine concentrations in µmol/l; median (p25-p75)									
	23 0/7 – 27 6/7w	n	28 0/7 – 31 6/7w	n	32 0/7 – 34 6/7w	n	35 0/7 – 36 6/7w	n	37 0/7 – 41 6/7w	n
M	3.07 (1.80 – 5.25)	21	1.53 (<0.1 – 2.19)	25	1.65 (0.31 – 2.28)	24	1.60 (<0.1 – 2.48)	25	4.71 (1.67 – 6.79)	27
CB	17.70 (11.22 – 28.67)	29	17.04 (9.79 – 20.26)	29	10.87 (6.56 – 15.91)	31	11.07 (8.06 - 15.09)	32	8.85 (6.79 - 12.55)	29
3d	10.60 (6.40 – 19.08)	28	7.68 (5.64 – 12.05)	30	8.33 (6.50 – 12.11)	32	9.87 (4.89 – 11.98)	31	8.12 (6.07 – 10.77)	29
14d	6.03 (4.80 – 8.39)	24	4.32 (3.16 – 5.95)	27	6.24 (3.72 – 7.31)	15	4.19 (0.64 – 7.69)	4	-	0
	Xanthine concentrations in µmol/l; median (p25-p75)									
M	0.09 (<0.03 – 0.25)	21	<0.03	25	<0.03 (<0.03 – 0.10)	24	<0.03 (<0.03 – 0.19)	25	0.18 (<0.03 – 0.25)	27
CB	0.47 (0.28 – 0.91)	29	0.54 (0.19 – 0.71)	29	0.32 (0.19 – 0.71)	31	0.34 (0.27 – 0.57)	32	0.43 (0.27 – 0.57)	29
3d	0.40 (0.23 – 0.86)	28	0.13 (<0.03 – 0.50)	30	0.38 (<0.03 – 0.76)	32	0.36 (<0.03 – 0.61)	31	0.47 (0.32 – 0.55)	29
14d	0.09 (<0.03– 0.35)	24	<0.03 (<0.03 – 0.26)	27	0.29 (<0.03 – 0.5)	15	0.16 (<0.03 – 0.39)	4	-	0

Table S 4 - Plasma hypoxanthine and xanthine concentrations (µmol/l) in the mothers (M), cord blood (CB) and the infants at 3 days (3d) and 14 days (14d) for each study group, shown as median (p25-p75), n= number samples analyzed

Table S5

	Adenosine concentrations in µmol/l; median (p25-p75)									
	23 0/7 – 27 6/7w	n	28 0/7 – 31 6/7w	n	32 0/7 – 34 6/7w	n	35 0/7 – 36 6/7w	n	37 0/7 – 41 6/7w	n
M	<0.01	21	<0.01	25	<0.01	24	<0.01	25	<0.01 (<0.01 – 0.01)	27
CB	<0.01 (<0.01 – 0.04)	29	<0.01 (<0.01 – 0.03)	29	<0.01 (<0.01 – 0.01)	31	<0.01 (<0.01– 0.02)	32	<0.01 (<0.01 – 0.01)	29
3d	0.05 (0.02 – 0.12)	28	0.02 (<0.01 – 0.03)	30	0.02 (<0.01 – 0.04)	32	<0.01 (<0.01 – 0.03)	31	0.01 (<0.01 – 0.02)	29
14d	0.03 (<0.01 – 0.05)	24	<0.01 (<0.01 – 0.02)	27	<0.01 (<0.01 – 0.02)	15	<0.01	4	-	0
	Inosine concentrations in µmol/l; median (p25-p75)									
M	<0.01 (<0.01 – 0.05)	21	<0.01	25	<0.01	24	<0.01	25	0.03 (<0.01 – 0.07)	27
CB	0.64 (0.26 – 3.78)	29	0.28 (0.16 – 0.74)	29	0.14 (0.07 – 0.35)	31	0.17 (<0.01 – 0.33)	32	0.12 (0.08 – 0.20)	29
3d	0.30 (0.15 – 0.70)	28	0.18 (0.10 – 0.26)	30	0.10 (<0.01– 0.33)	32	0.12 (<0.01 – 0.23)	31	0. 06 (<0.01 – 0.11)	29
14d	<0.01 (<0.01– 0.16)	24	<0.01	27	<0.01 (<0.01 – 0.09)	15	0.05 (0.01 – 0.08)	4	-	0

Table S 5 - Plasma adenosine and inosine concentrations (µmol/l) in the mothers (M), cord blood (CB), and the infants at 3 days (3d) and 14 days (14d) for each study group, shown as median (p25-p75); n= number samples analyzed

Table S6

	AMP concentrations in µmol/l; median (p25-p75)									
	23 0/7 – 27 6/7w	n	28 0/7 – 31 6/7w	n	32 0/7 – 34 6/7w	n	35 0/7 – 36 6/7w	n	37 0/7 – 41 6/7w	n
M	0.18 (0.12 – 0.31)	21	0.17 (0.14 – 0.27)	25	0.14 (0.08 – 0.17)	24	0.15 (0.07 – 0.32)	25	0.25 (0.11 – 0.31)	27
CB	0.59 (0.38 – 1.36)	29	0.50 (0.28 – 2.05)	29	0.26 (0.17 – 0.46)	31	0.36 (0.17 – 0.60)	32	0.33 (0.17 – 0.60)	29
3d	1.96 (1.15 – 3.95)	28	1.06 (0.71 – 1.71)	30	0.96 (0.79 – 1.50)	32	1.26 (0.68 – 2.16)	31	0.87 (0.58-2.12)	29
14d	1.36 (0.82 – 2.36)	24	0.85 (0.53 – 1.37)	27	0.71 (0.49 – 1.03)	15	1.20 (0.42 – 2.66)	4	-	0
	GMP concentrations in µmol/l; median (p25-p75)									
M	<0.01	21	<0.01	25	<0.01	24	<0.01 (<0.01 – 0.01)	25	<0.01	27
CB	<0.01 (<0.01 -0.06)	29	0.01 (<0.01 – 0.17)	29	<0.01 (<0.01 – 0.03)	31	<0.01 (<0.01 – 0.04)	32	<0.01 (<0.01 – 0.08)	29
3d	<0.01 (<0.01 – 0.14)	28	0.07 (<0.01 – 0.14)	30	0.05 (<0.01 – 0.11)	32	0.06 (<0.01 – 0.13)	31	0.08 (0.01 – 0.16)	29
14d	<0.01 (<0.01 – 0.05)	24	0.07 (<0.01 – 0.11)	27	<0.01 (<0.01 – 0.09)	15	<0.01	4	-	0

Table S 6- Plasma adenosine-monophosphate (AMP) and guanosine-monophosphate (GMP) concentrations (µmol/l) in the mothers (M), cord blood (CB), and the infants at 3 days (3d) and 14 days (14d) for each study group, shown as median (p25-p75), n= number samples analyzed

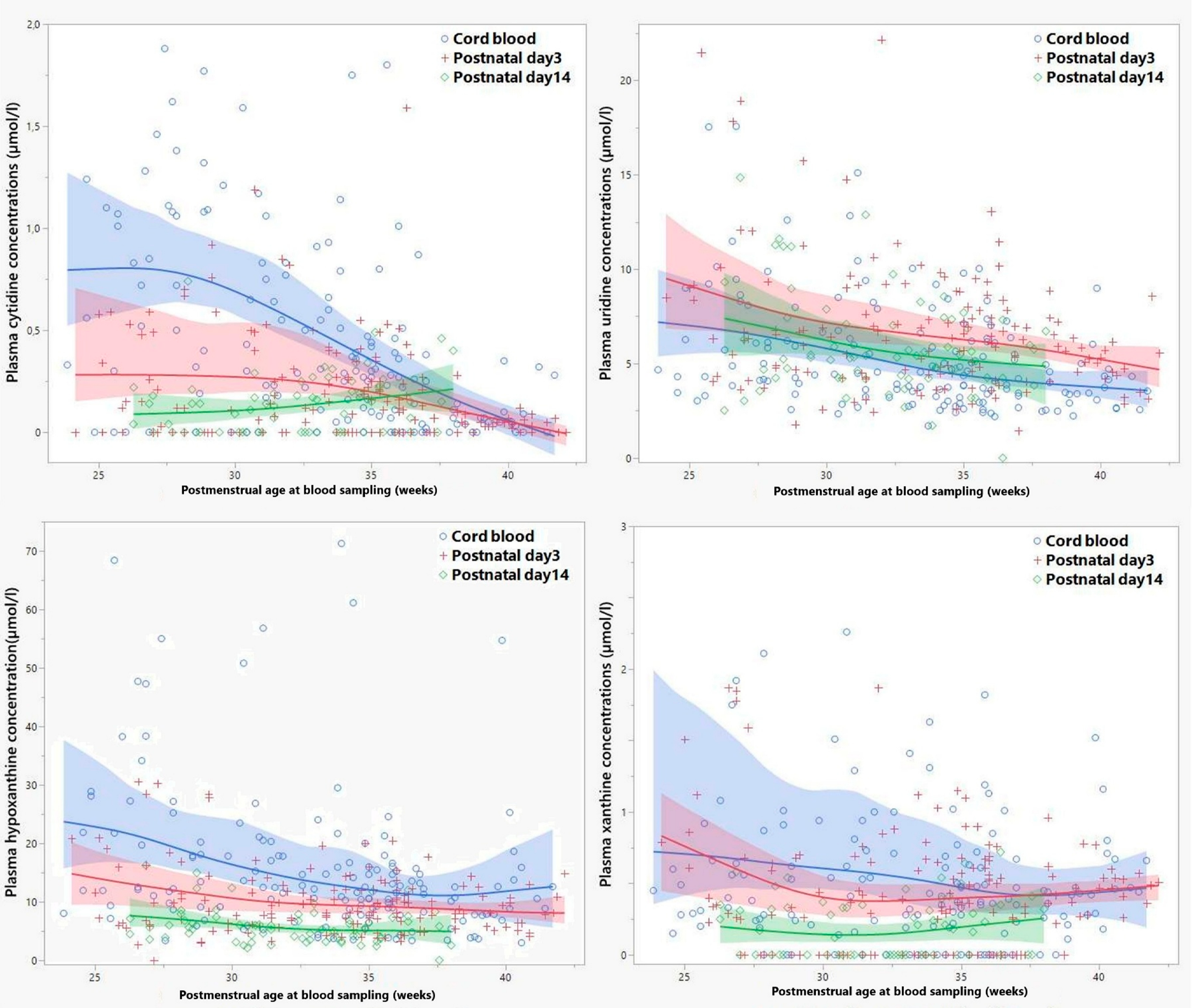

Figure S1a - Plasma concentrations (µmol/l) of cytidine, uridine, hypoxanthine, and xanthine according to postmenstrual age at blood sampling. Individual measurements are shown for cord blood, postnatal day 3, and postnatal day 14. Lines represent spline-smoothed median concentrations, with shaded areas indicating 95% confidence intervals. Colors and symbols distinguish the different sampling time points (blue = cord blood, red = postnatal day 3, green = postnatal day 14). Five observations (xanthine) are above the displayed y-axis. These observations were retained in the statistical analyses.

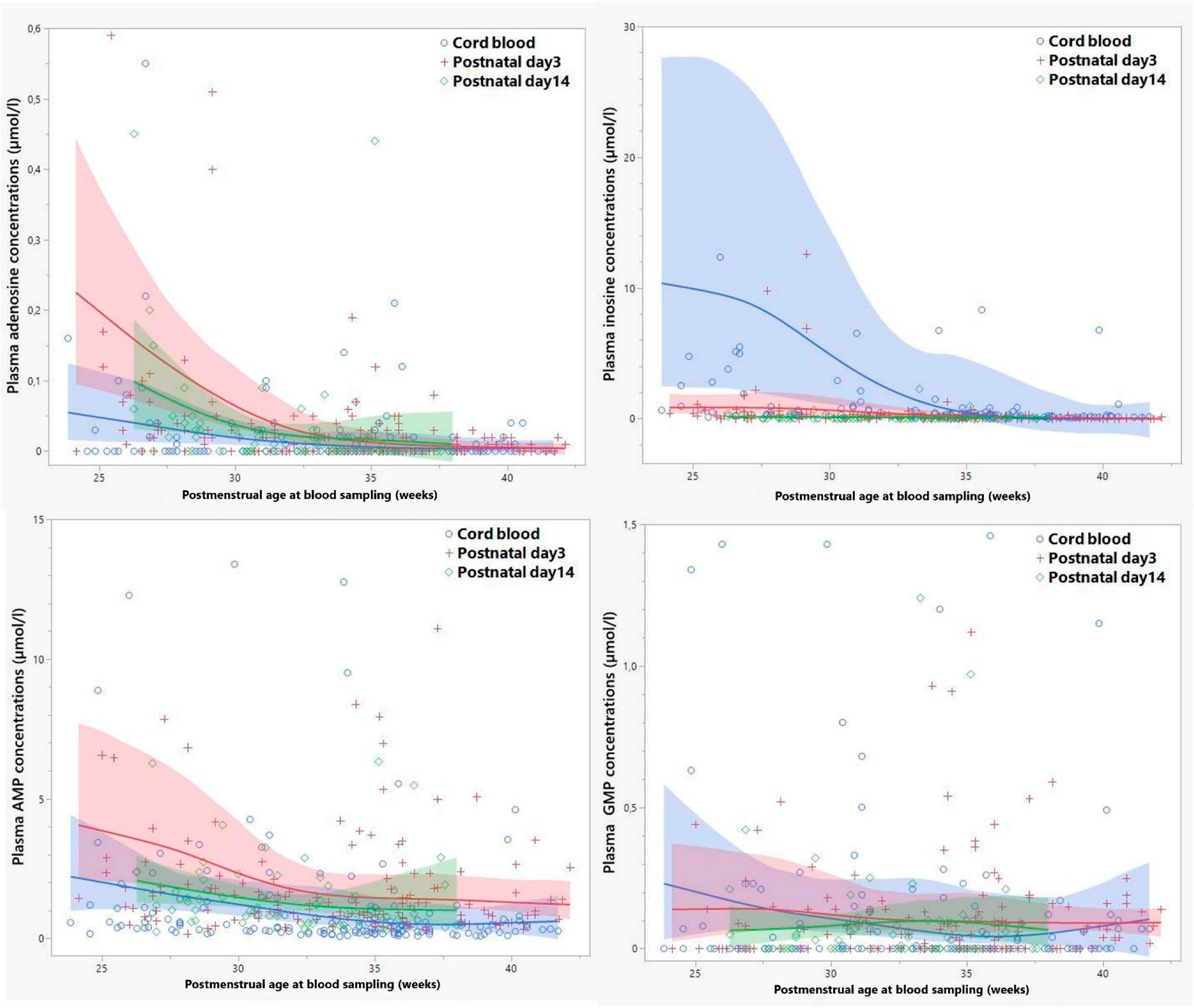

Figure S1b - Plasma concentrations (µmol/l) of adenosine, inosine, AMP, and GMP according to postmenstrual age at blood sampling. Individual measurements are shown for cord blood, postnatal day 3, and postnatal day 14. Lines represent spline-smoothed median concentrations, with shaded areas indicating 95% confidence intervals. Colors and symbols distinguish the different sampling time points (blue = cord blood, red = postnatal day 3, green = postnatal day 14). Two observations (adenosine), three observations (inosine), one observation (AMP) and two observations (GMP) are above the displayed y-axis. These observations were retained in the statistical analyses.
